# A Simple and Rapid Puncture Method for Draining Hematoma in Pontine Hemorrhage

**DOI:** 10.3389/fneur.2018.00794

**Published:** 2018-11-19

**Authors:** Mingzhe Zhang, Raynald Liu, Hongli Xing, Huixuan Luo, Leiting Cui, Zhaosheng Sun

**Affiliations:** ^1^Department of Neurosurgery, Harrison International Peace Hospital, Hebei Medical University, Hebei, China; ^2^Department of Neurosurgery, Beijing Neurosurgical Institute, Capital Medical University, Beijing, China; ^3^Raoyang County People's Hospital, Hebei, China

**Keywords:** pontine hemorrhage, puncture, method, rapid, simple

## Abstract

We present a patient with pontine hemorrhage. On admission, the patient was in a comatose state (Glasgow coma scale, 3). Due to rapid deterioration of his breathing, we immediately performed a direct puncture to the hematoma site. We present a simple and rapid puncture method for drainage of hematomas. The method is described and discussed in detail in this article. The described technique may be beneficial in emergency situations where the condition of the patient, particularly their respiration is declining rapidly.

## Introduction

Spontaneous brainstem hemorrhage is the most devastating type of intracranial hemorrhage (ICH). It accounts for 5–10% of ICH and primarily occurs in the pons (1–6). Delays in treatment are associated with high morbidity and mortality, with mortality rates ranging from 30 to 60% (7–9). Here, we report a case of spontaneous pontine hemorrhage. Due to an emergency situation, where the patient presented with respiratory dysfunction during admission, we performed an emergency hematoma evacuation via a puncture with a posterior approach. This method provides an effective, simple, and rapid procedure to relieve the pressure in the brainstem caused by a hematoma.

## Case illustration

A 50-year-old male was sent to our hospital due to sudden loss of consciousness, his Glasgow coma scale (GCS) score was 3 on admission. Neurological examination showed patient has no reaction to painful stimuli, fixed dilated pupils, fixed eyes in midposition without any ocular movement and no corneal reflexes on both eyes; the cranial nerves I, II, V, VII, VIII, and lower cranial nerves are unable to detect. Two hours before admission, the patient had experienced sudden palpitations and was sweating, he felt dizzy and suddenly lost consciousness. There was no nausea or vomiting prior to the onset of the symptoms. Head computed tomography (CT) revealed pontine hemorrhage. The patient had a history of hypertension for 10 years and coronary heart disease for 5 years and often did not take his medication regularly. On admission, there was a sudden decline in his breathing. We immediately performed tracheal intubation, and adjusted the ventilator to synchronized intermittent mandatory ventilation mode. We performed direct puncture of the pons to drain the hematoma. The patient's respiration subsequently improved and his GCS score recovered to 6. We adjusted the ventilator to continuous positive airway pressure mode. All procedures were completed within ~30 min. After the procedure, the fixed dilated pupils change into diminished response. However, the cranial nerves I, II, V, VII, VIII, and lower cranial nerves are still unable to detect. After the condition of the patient stabilize, he was transferred to another local hospital. Unfortunately, the patient died 1 month later, due to pulmonary infection.

## Puncture method

**Figure 1:**
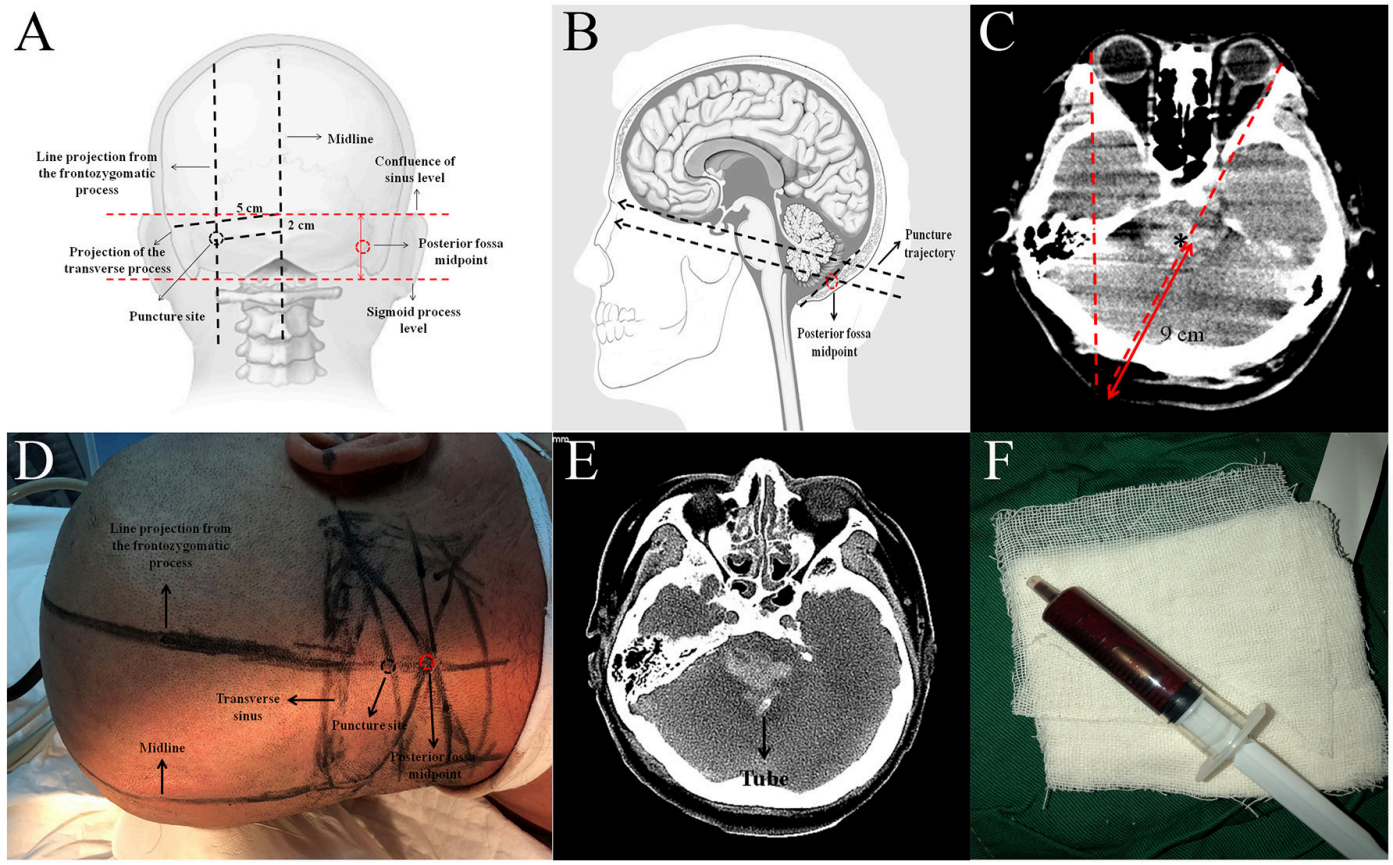
**(A)** Illustration of how to estimate the puncture site (adapted from www.LearnNeurosurgery.com). **(B)** Illustration of the puncture trajectory (adapted from www.daviddarling.info website). **(C)** Estimation of the direction and depth of the puncture. **(D)** Preoperative plan to estimate the puncture site. **(E)** Axial CT scan showing the tube within the hematoma region. **(F)** The hematoma extracted from the tube.

**Figure 2:**
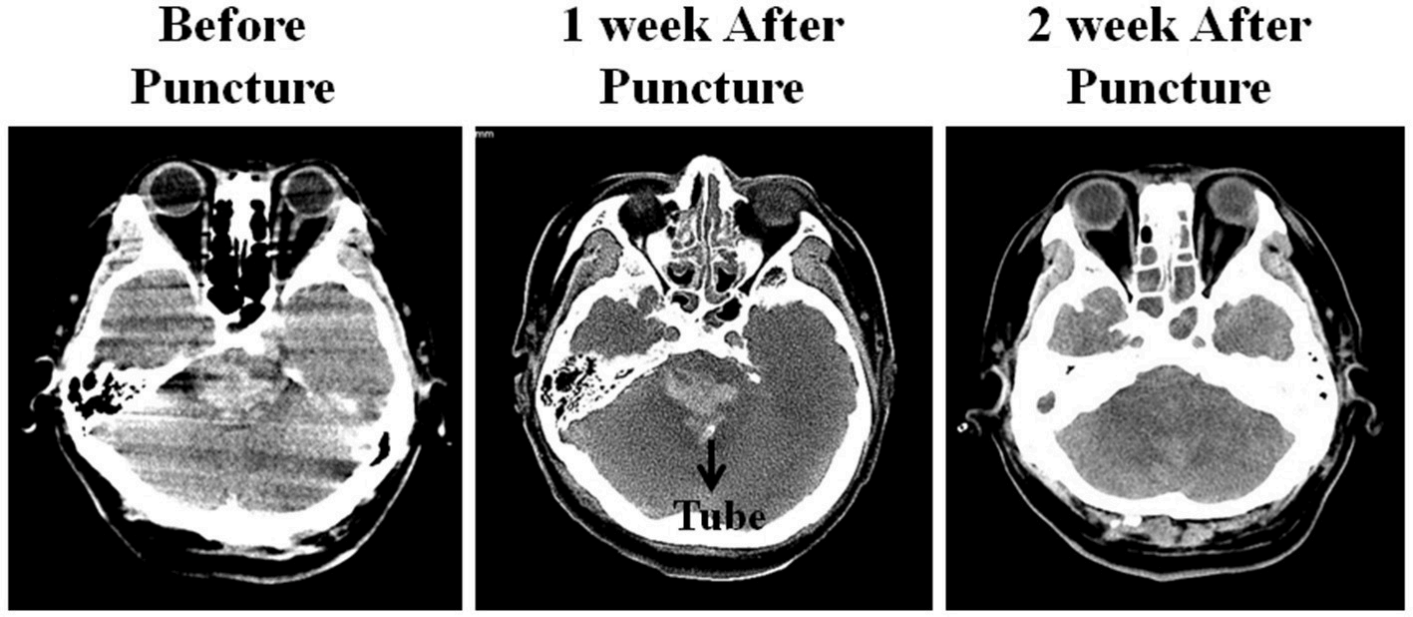
The hematoma condition showed on Head CT examination: **(A)** Before puncture, **(B)** no significant change of the hematoma 1 week after the procedure, **(C)** part of the hematoma was resolved 2 weeks after the procedure.

The puncture site was the intersection between a line projecting from the frontozygomatic process, which is ~± 5 cm from the posterior midline, and a horizontal line 2 cm below the transverse sinus. The posterior fossa midpoint was defined as the midpoint between the two transverse lines across the confluence of sinus and the sigmoid process (Figures 1A,D). The direction of the puncture in the axial plane was on the opposite side from the frontozygomatic process. The required depth of tube insertion was calculated from the skin to the midpoint of the hematoma (9 cm in this case) (Figures 1C,E). It should be noted that the puncture should be above the posterior fossa midpoint to ensure that it is not so low as to injure the medulla oblongata (Figure 1B). The bone above the posterior fossa midpoint is relatively flat, which facilitates generation of the puncture. When the tube was reaches the hematoma there will be a significant sense of breakthrough, and this feeling is highly important in indicating that the tube has reached the site of the hematoma. We extracted ~4.8 ml of the hematoma (Figure 1F). The volume of the hematoma was calculated at ~5 ml from the CT scan. We suggest that the volume of hematoma extracted should be less than the volume calculated. If there is any resistance during extraction, then the extraction should be stopped immediately. In our case, there was a reduction of the hematoma 2 weeks after the procedure (Figure 2).

## Discussion

Impaired consciousness is a major symptom associated with injury of the ascending reticular activating system (1, 3–5). Respiratory disturbances and failure, tetraplegia, decerebrate posture, hyperthermia, and pinpoint pupils are frequent signs of pontine hemorrhage (2, 4, 10). Among different types of intracranial hemorrhage, pontine hemorrhage is associated with unfavorable prognosis. Although patients may survive, various accompanying neurological sequelae are often unavoidable. Numerous studies have been conducted to identify prognostic factors in pontine hemorrhage. Various factors, including coma at admission, and location and volume of the hematoma, are believed to be associated with patient outcomes, although there remain many inconsistencies among published results due to the different parameters and populations included in analysis (11–14).

The outcome of hypertensive pontine hemorrhage is generally fatal, with rapid deterioration and death often occurring within hours (2, 15), hence, early treatment is necessary. With the advances in neuroimaging, CT and MRI can provide identification and precise localization of the brain stem lesions. In addition, CT guidance and localization techniques has provided safer method of the treatment for deep lesion in the brain (16). Several approaches have also been attempted for the posterior fossa lesions (17). In the past, stereotactic cerebral surgery was the only available procedure for pontine hemorrhage, despite its disadvantageous effects on maintenance of hemostasis and in causing injury to important tissue (16–18). The advances in neuroendoscopic techniques mean that this procedure can now be used as an alternative to stereotactic surgery (19–23). Takimoto et al. (24) reported a successful transaqueductal aspiration of a pontine hemorrhage with the aid of a neuroendoscope. Although neuroendoscopy is more complicated than stereotactic surgery, it has several advantages, including a reduction in the time required for target determination and body positioning, and the ability to perform concurrent treatment if there is also hydrocephalus.

In our case, due to rapid deterioration of the patient's condition, we performed a puncture to drain the hematoma, instead rather than open surgery to evacuate the hematoma. After we drained the hematoma, there was an improvement in the patient's breathing. Although we successfully delivered the tube to the hematoma site, this procedure is only recommended in cases where patient present with a rapid decline in their breathing. In cases that present with loss of consciousness alone, in the absence of respiratory disturbance, we did not recommended this procedure. Stereotactic surgery can provide accurate targeting of the puncture site; however, the simplicity of target determination and rapidity of the procedure we describe also provide several advantages in comparison with open, stereotactic, and endoscopic procedures. First, surgery to evacuate the hematoma, requires a skillful and trained surgeon, due to the complex anatomical structure and the high risk involved in brainstem surgery. Second, not all hospitals can provide stereotactic and endoscopic instruments. Third, preparation of surgical instruments and target determination is time-consuming. When the patient presents with respiratory disturbance, the brainstem cannot sustain more the increased compression caused by the hematoma. Therefore, we strongly recommended the use of this procedure for case of pontine hemorrhage with rapid respiratory deterioration. Although the patient died 1 month later from pulmonary infection, this puncture method was helpful in decompressing the brainstem, reversing the sharp decline in brainstem function with some slight improvement in the patient's immediate clinical status. We want to stress that our proposed intervention is not part of current guidelines for brainstem ICH. Therefore, the efficacy of the procedure requires additional verification, and comparative studies will be necessary in the future.

## Conclusions

Pontine hemorrhage is associated with high morbidity and mortality and early treatment is necessary to achieve an improved prognosis. Our puncture method provides a rapid and simple procedure for drainage of hematomas in the pons.

## Ethics statement

Written Informed Consent to publish the report was obtained from the patient's family. This study was approved by Institutional Review Board of Harrison International Peace Hospital, Hebei Medical University.

## Author contributions

Conception and design: MZ and RL. Drafting the article: RL. Acquisition of data, analysis and interpretation of data: HL, LC. Critical revision of the article: MZ, ZS and HX. Reviewed of the submitted version of the manuscript: all authors. Approved the final version of the manuscript on behalf of all authors: ZS.

### Conflict of interest statement

The authors declare that the research was conducted in the absence of any commercial or financial relationships that could be construed as a potential conflict of interest.
